# Does exposure to new transport infrastructure result in modal shifts? Patterns of change in commute mode choices in a four-year quasi-experimental cohort study

**DOI:** 10.1016/j.jth.2017.07.009

**Published:** 2017-09

**Authors:** Eva Heinen, Amelia Harshfield, Jenna Panter, Roger Mackett, David Ogilvie

**Affiliations:** a MRC Epidemiology Unit and UKCRC Centre for Diet and Activity Research (CEDAR), University of Cambridge, Box 285, Cambridge Biomedical Campus, Cambridge CB2 0QQ, United Kingdom; b University of Leeds, Institute for Transport Studies, Faculty of Environment, LS2 9JT Leeds, United Kingdom; c The Primary Care Unit, Institute of Public Health, University of Cambridge, Box 113 Cambridge Biomedical Campus, Cambridge, CB2 0SR, United Kingdom; d Centre for Transport studies, University College London, Gower Street, London WC1E6BT, United Kingdom

## Abstract

**Background:**

Intervention studies suggest that changing the built environment may encourage a modal shift from car travel towards active travel. However, little is known about the detail of patterns of changes in travel behaviour.

**Method:**

Adult commuters working in Cambridge (UK) completed annual questionnaires between 2009 and 2012. Commuting was assessed using a validated seven-day travel-to-work record. The intervention consisted of the opening of a guided busway with a path for walking and cycling in 2011. Exposure to the intervention was defined as the negative of the square root of the shortest road distance from home to the busway. We investigated the association between exposure to the intervention and specific modal shifts and patterns of change, along with individual mode choice patterns over the entire four-year period.

**Results:**

Five groups of patterns of change were found in our in-depth explorations: (1) no change, (2) a full modal shift, (3) a partial modal shift, (4) non-stable but patterned behaviour, and (5) complicated or apparently random patterns. A minority of participants had a directed change of either a full modal shift or, more commonly, a partial modal shift, whereas a large proportion showed a highly variable pattern. No significant associations were found between exposure to the intervention and specific modal shifts or patterns of change.

**Conclusion:**

Our analyses revealed a large diversity in (changes in) travel behaviour patterns over time, and showed that the intervention did not result in one specific pattern of behaviour change or produce only full modal shifts. These insights are important for improving the measurement of travel behaviour, improving our understanding of how changes in travel behaviour patterns occur, and fully capturing the potential impacts of interventions.

## Highlights

• We explored individual mode choice patterns in a 4-year cohort.
• Our analyses showed large variation in (changes in) travel behaviour patterns.
• A partial modal shift was more common than a full modal shift.
• A large proportion of our cohort showed a highly variable pattern.
• Our findings will help in fully capturing the potential impacts of interventions.

## 1 Introduction

Active travel can provide a sufficient level of physical activity to improve health and well-being (Department of Health, 2011). People who walk or cycle to work have been found to have lower cardiovascular risk than individuals who do not. Active travel may particularly be beneficial in the form of commuting as it is a repetitive journey and relatively easily incorporated in daily life (Hamer and Chida, 2008). As a result, modal shifts towards active travel are often encouraged.

Several recent studies have investigated patterns of travel behaviour change using panel data. For example, Clark et al. (2016) explored the prevalence and predictors of changes in commute mode choice in the United Kingdom Household Longitudinal Study. They showed that a third of the individuals who cycled or used the bus to travel to work, a quarter of those who walked, and a tenth of those who drove to work in 2009/10 had changed their commute mode in the next annual survey. These changes appeared to be primarily driven by changes in commute distance. Another study by Panter et al. (2011) investigated changes in commute mode choice among 655 commuters in Cambridge, UK between 2009 and 2010, and tested the socio-economic, spatial and psychological predictors of changes in time spent cycling and walking for commuting purposes in a one-week period. The majority of the participants reported the same usual mode of transport in both waves, whereas 6% of the respondents switched to the car in the given timeframe and a similar share switched away. The average duration of walking for commuting purposes increased by 3 min, and the average time cycling decreased by 5.3 min. The changes in time spent active commuting were largest in those groups who had shifted towards or away from the car use as the mode of transport.

Other studies have focussed more on the variability of individual travel behaviour and transitions within the mixture of modes used. Kroesen (2014), for example, investigated the predictors of transitions between data-derived travel behaviour clusters using latent class and latent transition models and showed that individuals who used multiple modes were more likely than those who relied on a single mode to change from one travel behaviour cluster to another over time. Chatterjee et al. (2016) also analysed transitions in commute mode choice patterns using one-week travel data collected every three-months. They found large variations between individuals in terms of their commute patterns. Almost 40% of the respondents only used a car, a similar percentage never used a car, and the remainder sometimes used a car. The data also revealed the presence of some transitions in car use, in which most changes occurred towards or away from occasional car use and fewer between the two more ‘extreme’ groups. These studies show that, over time, changes in commute mode choice may occur ‘naturally’, potentially driven by life events. However, they have not investigated changes resulting from interventions, nor have they provided a full picture of all individual mode choices in the modal mix and the transitions within them.

One approach to stimulating a shift towards active travel is to make the built environment more supportive for the use of these modes of transport. Cross-sectional studies have found associations between the built environment and mode choice (e.g. Handy et al., 2005; Ewing and Cervero, 2010; Handy et al. 2002; Saelens et al., 2003). More recently, several natural experimental studies have provided some evidence that interventions in the built environment, such as the introduction of new high-quality infrastructure, may encourage an increase in active travel. Heinen et al. (2015) showed that the opening of a guided busway, a separate guided track for buses which allows them to reach a high speed with an adjacent high quality walking and cycling path, produced a change in commute mode choice among those living nearby, in that individuals who lived closer to the new infrastructure were more likely to reduce their car use and more likely to increase their share of trips involving active travel. Using the same data, Panter et al. (2016) showed that individuals who lived closer to the intervention were also more likely to increase the time spent cycling in their weekly commutes. Goodman et al. (2014) analysed the impact of new walking and cycling routes on 1796 residents in three municipalities in the UK. They concluded that proximity to new infrastructure did not predict change in active travel after one year. However, after two years, living closer to the new infrastructure predicted an increase in weekly minutes spent walking and cycling.

Although these studies provide new support to the hypothesis that interventions in the built environment can change travel behaviour in a societally favourable direction, the patterns of change have not yet been explored in detail. The measured associations may be a result of small changes in travel behaviour in most individuals, or may be a result of large changes of fewer individuals. Moreover, the existing studies mainly show whether modal shifts took place towards (or away from) active travel. Changes in travel behaviour for individuals whose active travel mode share or active travel time remained stable are mostly unexplored. To illustrate this with an example: suppose an individual changes from commuting by bicycle only to the combined use of public transport and walking. This change may not necessarily affect the overall active commuting time, neither would it result in a change in the share of trips involving active travel. Nonetheless, large changes in individual travel behaviour *have* occurred in terms of (a) the primary mode (from bicycle to bus) as well as (b) the form of active travel (from cycling to walking). Neither of these changes are fully captured in most existing intervention studies. It is important to glean a deeper understanding of such changes in order to project consequent impacts (e.g. on air pollution, congestion or physical activity) and longer-term behavioural changes (e.g. the development or breaking of habits, self-efficacy to use certain modes) as well as to estimate the potential modal shift impacts of similar interventions in the future.

In this paper, we will characterise patterns of change over time using data from the four-year *Commuting and Health in Cambridge* quasi-experimental study cohort, and test whether exposure to the intervention is associated with certain individual behavioural patterns. We will pay particular attention to all modes used in the modal mix and explore the modal shifts, the patterns of mode choice and the patterns of changes in mode choice. To this end, we will analyse individual one-week commuting records collected annually over four years. This will allow us to explore shorter- and longer-term patterns of behaviour in general, as well as the modal shifts associated with a particular intervention.

## 2 Method

### 2.1 Setting and study: Cambridgeshire Guided Busway

The *Commuting and Health in Cambridge* study aimed to understand the impacts of a major transport infrastructural intervention in Cambridgeshire, UK, on travel behaviour, physical activity and related health outcomes (Ogilvie et al., 2016). The city of Cambridge (123,900 inhabitants) has a comparatively affluent and well-educated population. In 2011, 45% of its commuting population travelled to work by car or taxi, 28% by bicycle, 15% on foot, and 9% by public transport (ONS, 2011).

The physical intervention comprised the Cambridgeshire Guided Busway, a 25-km guideway (separate off-road track) for specially adapted buses, with a parallel service path that offers high-quality segregated infrastructure for non-motorised transport such as walking and cycling. The busway connects the city centre and several major employment sites with surrounding towns and villages, and was opened in 2011. The principal aim of the busway was to change travel behaviour in order to reduce traffic congestion (Atkins, 2004, British Broadcasting Corporation (BBC), 2014) by offering improved facilities for public transport, walking, and cycling.

### 2.2 Data collection and study sample

Questionnaire data were collected by post annually between 2009 and 2012 as part of a natural experimental cohort study. At the time of recruitment, participants were 16 years of age or over, working in areas of Cambridge to be served by the busway and living within approximately 30 km of the city centre (Ogilvie et al., 2016). To avoid biasing recruitment and responses, the study was presented to participants as a study of ‘commuting and health’ and the aim of evaluating the busway was not made explicit. The study was not designed to recruit a sample representative of the population of the city or its region.

**Fig. 1:**
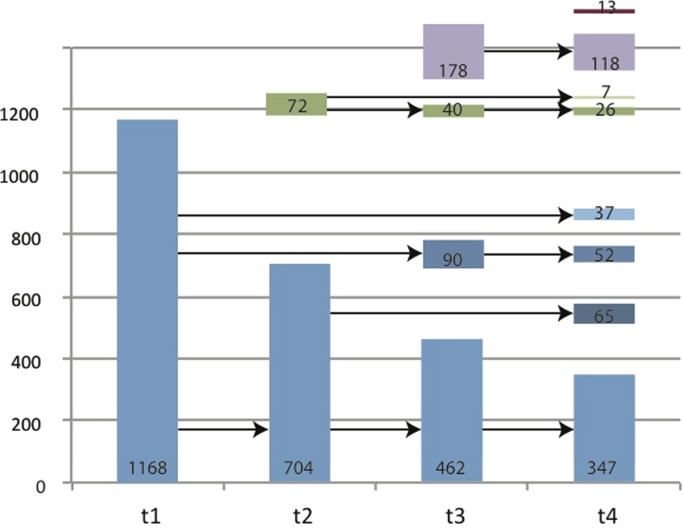
Respondents over time*. *The four waves of data collection are represented on the x-axis. The flow of the participants is illustrated through the waves. Participants added in later waves are represented using different colours. All shades of blue represent participants who began in 2009; green, those who began in 2010; and purple, those who began in 2011 or 2012. (For interpretation of the references to color in this figure legend, the reader is referred to the web version of this article.)

The busway was implemented during the second and part of the third waves of data collection. Its construction began in March 2007 and it was opened on 7 August 2011, more than two years later than planned. However, the busway could be accessed and used unofficially at several locations before the official opening, especially for walking and cycling. Therefore, the first wave can be considered entirely pre-intervention and the fourth wave entirely post-intervention. 1164 participants took part in the first wave. Of these, 704, 462, and 347 participants continued in the second, third, and fourth waves, respectively. The cohort was ‘topped up’ in each wave, resulting in a total of 1168 individuals who participated in the first wave, 776 in the second, 770 in the third, and 565 in the fourth wave (Fig. 1). The analyses reported in this paper comprise those participants who participated in all four survey waves (n = 347).

The Hertfordshire Research Ethics Committee approved the study as well as the baseline (Reference no.: 08/H0311/208), second (09/H0311/116) and third (10/H0311/65) data collections of the cohort study. The Cambridge Psychology Research Ethics Committee approved the final data collection used in this analysis (Reference no.: 2014.14). All participants provided written informed consent.

### 2.3 Exposure

To understand the effect of the intervention, an exposure measure was derived based on the proximity of the home postcode of each participant at baseline to the busway stop or path access point, whichever was nearest (Heinen et al., 2014). We applied a negative square root transformation to the distance, so that greater proximity corresponded with a higher level of exposure to the intervention, as in previous published analyses of the data (Heinen et al., 2014, Heinen et al., 2015, Panter et al., 2016, Prins et al., 2016).

### 2.4 Outcome

Individual travel behaviour was measured by a validated (Panter et al., 2014) self-reported seven-day commute travel diary in each wave. For each day, respondents were asked to report the day of the week, their working hours and their mode(s) of travel to and from work, or to positively indicate that they had not travelled to work that day. The respondents were explicitly encouraged to report all travel modes used for each trip. No imputations or deletions were made, even if travel data appeared incomplete or incorrect.

**Fig. 2:**
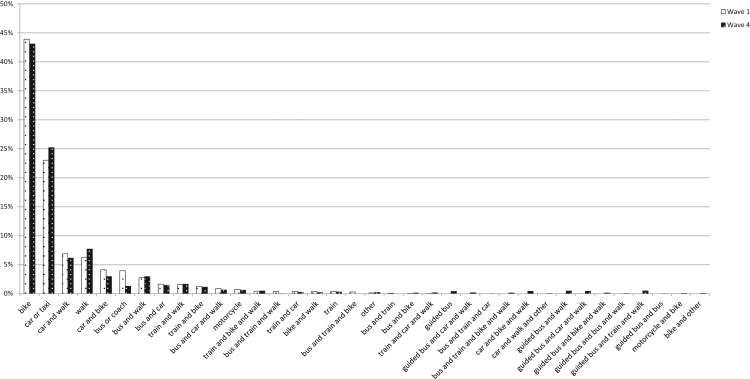
Modal shares in waves one and four of individuals participating in both waves.

Fig. 2 presents the mode shares in wave one in white and wave four in black. In our cohort, the bicycle was the most commonly used commute mode before as well as after the intervention, followed by car/taxi. Only walking and the combination of car and walking also had mode shares over 5%. Fig. 2 also illustrates that a large variety of (combinations of) modes was used.

Changes in the aggregate mode share of the cohort over time were small. Car use increased the most, by more than two percent points, followed by walking. The mode shares for conventional bus (which was distinguished from guided bus in data collection) and the use of the train in combination with the bicycle fell by more than one percentage point. The percentage of trips made by bicycle also decreased slightly.

### 2.5 Analyses

We conducted person-level analyses, first summarising each person's mode choice behaviour at trip level, always taking all the modes used in one trip into account, and then analysing individuals’ weekly mode choice patterns over time. We explored individual patterns of travel behaviour and changes in these patterns in two analyses.

#### 2.5.1 Analysis 1

We explored individual travel patterns over time in depth. All individuals who participated in all four waves were included. After exploring the patterns quantitatively, we used a more descriptive approach and colour-coded individual mode choices per trip to confirm and further our understanding of the patterns, and finally to assign individuals to a specific pattern. Each mode or combination of modes used per trip was identified by a unique colour. We visualized the patterns of mode choice, and as a result groups of individuals with five distinct patterns of mode choice over time emerged.

#### 2.5.2 Analysis 2

We conducted a person level analysis and tested whether an association existed between exposure to the intervention and the patterns of behaviour revealed in analysis 1. We then tested whether individuals who were more exposed to the guided busway were more likely to have a full or partial modal shift or another change in their travel behaviour. For this we estimated a multinomial regression model in which ‘no change’, i.e. a stable behaviour pattern over time, served as the reference category. The following additional covariates were considered: gender, age, commute distance, the availability of (free) parking at work, education level, car ownership, access to a bicycle, housing tenure, presence of children in the household, presence of a limiting long-term health condition, difficulty walking, moving working or residential location, and residential settlement size (the Urban Rural Classification of the Census Output Area) (Bibby and Shephard, 2004), all assessed at baseline. Each covariate was tested independently on every outcome variable and only adjusted for if p < 0.25 in the unadjusted models. We defined individuals as having ‘no change’ if they did not change their pattern of mode use over the waves.

We defined a full modal shift as a change of modes between the first wave and the fourth wave in this analysis. A partial modal shift was defined as a partial change of commute modes between waves that was not reversed in later waves. The difference between a full modal shift and a partial shift is that the mode of travel did not change in all commute trips.

## 3 Results

### 3.1 Analysis 1: Patterns of change

Gaining a better insight into the patterns of change may contribute to an improved understanding of the full effect of the intervention. We firstly present analyses by mode (3.1.1, 3.1.2, 3.1.3), followed by an analysis of the full modal mix (3.2, 3.3).

#### 3.1.1 Patterns of car use

Many respondents showed similar patterns of behaviour in terms of car use over the four waves (Appendix A). Approximately 20% of the cohort always used a car for the entire commute trip in all four waves, and 20% did not use a car at all. Nevertheless, a larger share of the population showed a more complicated pattern. About 8% of our respondents showed a change from using the car as the only mode of transport in all trips, to a pattern of less frequent or less ‘intense’ car use. The opposite trend was, however, more prevalent: a proportion of respondents increased their intensity of car use by changing from partly using the car to driving the entire journey to/from work, and another group changed from being non-drivers to occasional drivers.

#### 3.1.2 Patterns of active travel

Almost 75% of the respondents travelled by active travel in at least one of the waves (Appendix B). Approximately 20% made a trip entirely by an active travel mode (and not in combination with other modes) in the first wave and continued this behaviour in consequent waves. Cycling appeared to be more commonly used for the entire trip, whereas walking was mostly done in combination with other modes of transport.

#### 3.1.3 Patterns of public transport use

Public transport use in our cohort was more limited than car use and active travel (Appendix C). Only a small percentage of the respondents used public transport in all four waves, and only approximately one third of the respondents used public transport for commuting purposes in any wave.

### 3.2 Patterns of modal shift

**Fig. 3:**
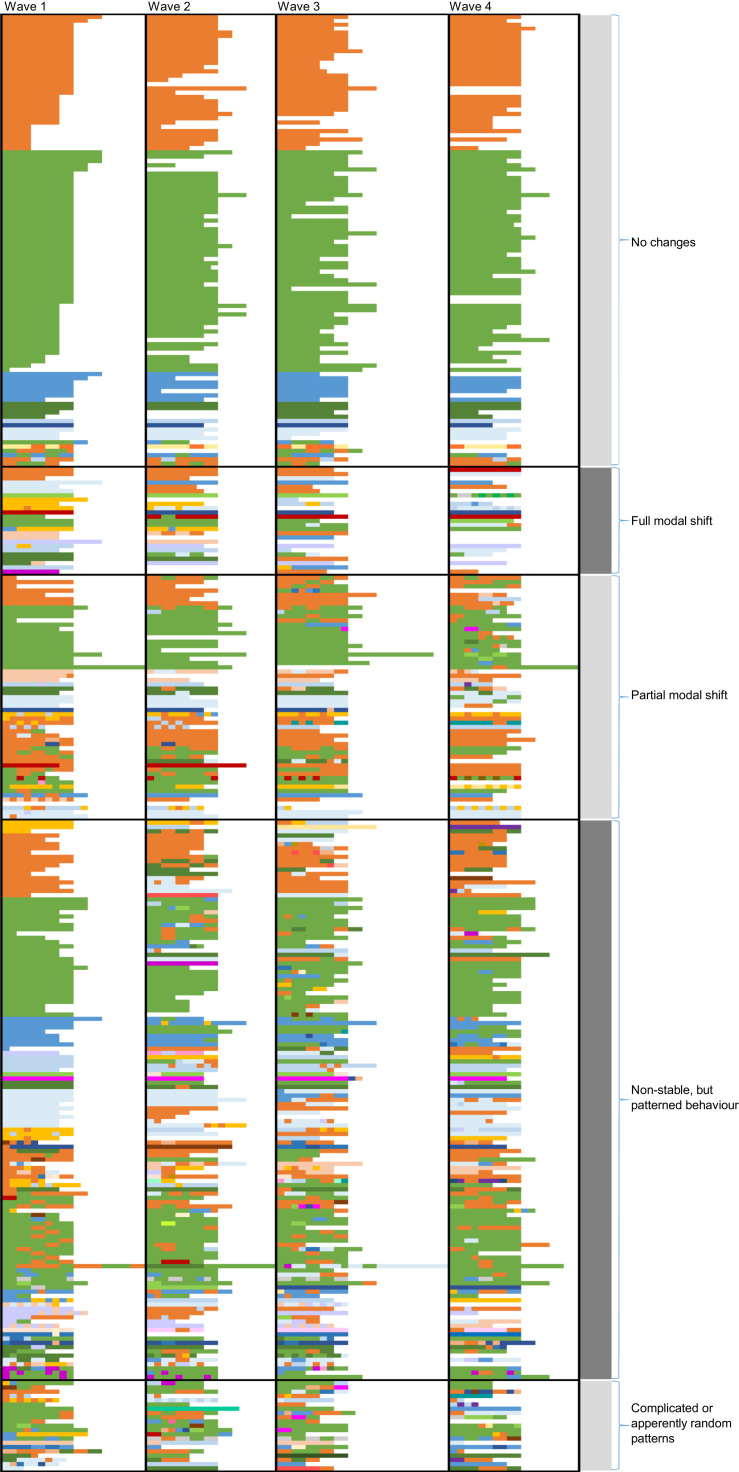
Overview of all mode choices over the four waves. Each horizontal line represents an individual. The waves are separated by vertical black lines. Each trip is one square, individually colour-coded and the length of the line corresponds with the number of commute trips made over one week. Each colour represents one modal combination. Orange = car only; green = bicycle only; blue = walk only; yellow = train only; and brown = bus only. The white colour indicates no trip was made. Full legend: see Appendix D. (For interpretation of the references to color in this figure legend, the reader is referred to the web version of this article.)

Fig. 3 shows the patterns of change in mode choice in individuals over time. Each transportation mode or combination of modes was assigned a unique colour (orange = car, green = bicycle, blue = walk, yellow = train, and brown = bus). Given the large combination of potential combination of modes within a trip, not all colours are discussed in detail, but the aim was to provide similar colours to modal combinations that share similarities (for the full legend: see Appendix D). The visual analysis revealed five patterns of change over time: (1) no change, (2) a full modal shift, (3) a partial modal shift, (4) non-stable but patterned behaviour, and (5) complicated or apparently random patterns. We will now discuss these patterns in greater detail.

#### 3.2.1 Group 1: No changes

**Fig. 4:**
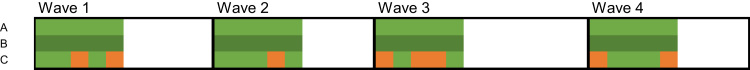
Examples of patterns of no change. Each horizontal line is an individual. The waves are separated by vertical black lines. Each trip is one square, individually colour-coded and the length of the line corresponds with the number of commute trips made over one week. Each colour represents one modal combination. Orange = car only; green = bicycle only; blue = walk only; yellow = train only; and brown = bus only. The white colour indicates no trip was made. Full legend: see Appendix D. (For interpretation of the references to color in this figure legend, the reader is referred to the web version of this article.)

A large proportion, one third, of respondents did not change their pattern of mode use over the waves. These respondents may be separated further into two groups. One group consisted of individuals who showed no variation at all. For example, individual A in Fig. 4 cycled all their commute trips in all four waves. A smaller proportion of respondents also showed no variation over time, but they used a combination of modes for each trip, for example car and bicycle (individual B in Fig. 4).

The second group of participants that could be distinguished were those respondents whose pattern did not change, but who used different transport modes for different trips. For example, individual C in Fig. 4 varied their mode of transport day-to-day between car and bicycle. This group did not necessarily maintain similar mode shares over time. For example, Fig. 4 shows individual C whose car share varied over the waves from 40% in waves 1 and 4, 20% in wave 2 and 60% in wave 3, and the remaining trips were all made by bicycle. Nevertheless, their patterns remained similar.

#### 3.2.2 Group 2: A full modal shift

**Fig. 5:**
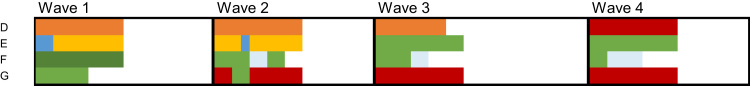
Examples of full modal shift patterns. Each horizontal line is an individual. The waves are separated by vertical black lines. Each trip is one square, individually colour-coded and the length of the line corresponds with the number of commute trips made over one week. Each colour represents one modal combination. Orange = car only; green = bicycle only; blue = walk only; red = motorcycle; yellow = train only; and brown = bus only. The white colour indicates no trip was made. Full legend: see Appendix D. (For interpretation of the references to color in this figure legend, the reader is referred to the web version of this article.)

In our cohort, a group of individuals – approximately 8% of the cohort – could be identified who showed a full modal shift between waves. Most often, it is assumed in a full modal shift that one transfers from one mode to another, as illustrated by individual D in Fig. 5. However, we also identified three other types of full modal shift. For example, some individuals used two methods of travel before the intervention and changed to a third mode/combination of modes after the intervention (individual E in Fig. 5). We also identified examples of the opposite pattern: from one mode (or combination of modes) to two other ways of travel (individual F in Fig. 5). A final example of a full modal shift is an individual who initially showed a partial shift, before shifting entirely (individual G in Fig. 5). This pattern was present in only a few individuals.

No specific modal shift occurred regularly when looking at all four waves. When we focus on all full modal shifts that took place when only considering waves 1 and 4 (and the corresponding sample, i.e. n = 501), some more common full modal shifts were observable. Ten individuals shifted from the bicycle to the car or taxi, whereas 12 individuals showed the opposite shift. Other notable shifts included eight individuals who exchanged (part of their trip by) a conventional bus for the guided bus, and two who shifted from car to guided bus. Other full shifts were observed but were less prevalent.

#### 3.2.3 Group 3: A partial modal shift

**Fig. 6:**
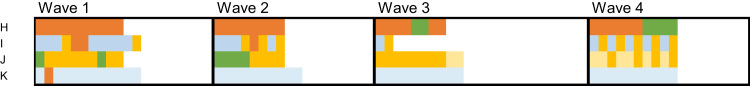
Examples of partial modal shift patterns. Each horizontal line is an individual. The waves are separated by vertical black lines. Each trip is one square, individually colour-coded and the length of the line corresponds with the number of commute trips made over one week. Each colour represents one modal combination. Orange = car only; green = bicycle only; blue = walk only; yellow = train only; and brown = bus only. The white colour indicates no trip was made. Full legend: see Appendix D. (For interpretation of the references to color in this figure legend, the reader is referred to the web version of this article.)

Approximately 16% of our respondents showed a partial modal shift, a larger group than the group that showed a full modal shift. Fig. 6 illustrates several examples of such partial shifts. Individual H in Fig. 6 added an additional mode of commuting over time – the ‘simplest’ form of a partial modal shift. This pattern was present in our cohort with changes in every wave. In addition, there was a group of individuals who either added a mode of transport to a more complicated commute pattern, or dropped one mode (individual I in Fig. 6). Another common partial modal shift was that of an individual who used two modes to commute and substituted one of these with another mode of transport (individual J in Fig. 6). The individual shown in the example changed from commuting sometimes by bicycle and sometimes by bus, to travelling sometimes by bus and sometimes by guided bus. Finally, a number of individuals in our cohort had one or two trips in the first or last wave that were different (individual K in Fig. 6).

#### 3.2.4 Group 4: Non-stable but patterned behaviour

**Fig. 7:**
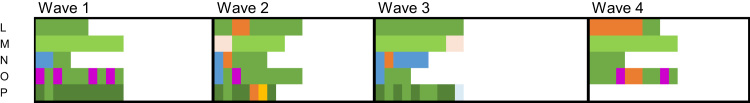
Examples of non-stable but patterned behaviour. Each horizontal line is an individual. The waves are separated by vertical black lines. Each trip is one square, individually colour-coded and the length of the line corresponds with the number of commute trips made over one week. Each colour represents one modal combination. Orange = car only; green = bicycle only; blue = walk only; yellow = train only; and brown = bus only. The white colour indicates no trip was made. Full legend: see Appendix D. (For interpretation of the references to color in this figure legend, the reader is referred to the web version of this article.)

A large proportion of the cohort (approximately 38%) showed non-stable behaviour over the waves that could not be classified as a modal shift. The majority of these respondents, however, showed similarities in their behaviour patterns in the different waves. For example, Fig. 7 shows individual L whose commute mode choices in waves 2 and 4 differed from those in waves 1 and 3. In all waves, this person cycled the entire trip to work on at least some days, but in two of the waves also drove to work for some of their trips. A similar pattern of behaviour is illustrated by individual M in Fig. 7. The travel behaviour of this individual in waves 2 and 3 differed from their behaviour in the first and last wave. More complicated patterns are illustrated by individuals N and O in Fig. 7. Individual N initially had a modal mix with some walking and some cycling trips. In the second wave, they added the car to this mix. In the third wave, this individual mostly walked and made only one trip by car, before returning to cycling in the last wave. Individual O in Fig. 7 sometimes commuted entirely by bicycle, and sometimes by a combination of walking and cycling in wave 1. In the second wave, one commute trip was made entirely by walking and the remaining trips were fairly similar to the first wave. A similar pattern emerged in wave 3, but in the final wave, two trips were made by car. Finally, individual P in Fig. 7 showed a partial shift between waves 1 and 2. In wave 3, they shifted back to an almost identical original pattern as in wave 1, but added yet another mode.

#### 3.2.5 Group 5: Complicated or apparently random patterns

**Fig. 8:**
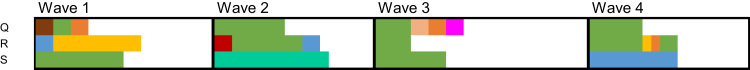
Examples of complicated or apparently random patterns. Each horizontal line is an individual. The waves are separated by vertical black lines. Each trip is one square, individually colour-coded and the length of the line corresponds with the number of commute trips made over one week. Each colour represents one modal combination. Orange = car only; green = bicycle only; blue = walk only; yellow = train only; and brown = bus only. The white colour indicates no trip was made. Full legend: see Appendix D. (For interpretation of the references to color in this figure legend, the reader is referred to the web version of this article.)

Twenty individuals in the cohort (approximately 6%) showed a very complicated pattern of behaviour, which was too variable between waves to be assigned to one of the previously identified groups. For example, individual Q in Fig. 8 used a train for some commute trips, a bicycle for other trips, and a car for their remaining trips in wave 1. They used only a bicycle in wave 2. This was followed by the use of a bicycle for some trips, a car and train for other trips, a car only for two trips, and a train, bike and walk for the remaining two commute trips in wave 3. In the last wave, they solely commuted by bicycle. Individuals R and S in Fig. 8 are examples of individuals with slightly less complicated patterns, but they still do not show a structured behaviour pattern.

### 3.3 Analysis 2: Predictors of individual modal shift

We tested whether exposure to the intervention was associated with patterns of individual mode choice behaviour as revealed in analysis 1. First, we explored the membership characteristics of each group (Table 1). There were differences between the characteristics of the members of the groups. For example, compared to the other groups, a larger percentage of the members of group 5, individuals with ‘complicated or apparently random patterns’, were over sixty years old. Perhaps most notable was the fact that the two groups that represent a full modal shift (Group 2) and a partial modal shift (Group 3) included fewer individuals living in urban settings. Nevertheless, most differences were small and no group differed in all aspects from other groups.

**Table 1:** Characteristics of members in each pattern.

		Group 1	Group 2	Group 3	Group 4	Group 5
Exposure^a^		− 2.12 (1.44)	− 2.70 (1.36)	− 2.10 (1.32)	− 2.18 (1.49)	− 1.94 (1.38)
Age	Under 30 (n = 32)	8.4%	7.0%	7.1%	11.4%	9.5%
	30–40 (n = 79)	23.4%	34.6%	28.6%	18.9%	19.1%
	40–50 (n = 100)	29.0%	26.9%	30.4%	29.6%	28.6%
	50–60 (n = 100)	28.0%	30.8%	28.6%	31.1%	23.8%
	Over 60 (n = 31)	11.2%	0.0%	5.4%	9.1%	19.1%
Gender	Male (n = 113)	35.8%	30.8%	41.1%	28.0%	28.6%
	Female (n = 231)	64.2%	69.2%	58.9%	72.0%	71.4%
Type of settlement	Urban (> 10,000) (n = 229)	72.5%	57.7%	55.4%	65.9%	81.0%
	Town & Fringe (n = 57)	13.8%	15.4%	25.0%	16.7%	9.5%
	Village, Hamlet & Isolated Dwellings (n = 58)	13.8%	26.9%	19.6%	17.4%	9.5%

Group 1: No change; Group 2: Full modal shift; Group 3: Partial modal shift; Group 4: Non-stable, but patterned behaviour; Group 5: Complicated or apparently random patterns.

a Reported are the mean values and standard deviations.

Second, we tested whether exposure to the intervention was associated with the individual patterns of mode choice behaviour as revealed in analysis 2, whilst controlling for covariates.

Individuals who were more exposed to the intervention were not more likely to have a full or partial modal shift (Table 2). In neither the unadjusted nor the maximally adjusted models was a significant association (at the 95% confidence level) found between exposure to the intervention and any specific pattern of behaviour.

**Table 2:** Associations between exposure to the intervention and patterns of change.

	**Unadjusted model**			**Maximally adjusted model**		
	**RRR**	**95% CI**	**P-value**	**RRR**	**95% CI**	**P-value**
**Full modal shift**	0.77	[0.58–1.02]	0.07	0.83	[0.60–1.16]	0.27
**Partial modal shift**	1.01	[0.80–1.27]	0.95	1.12	[0.86–1.46]	0.40
**Patterned, but non-stable behaviour**	0.97	[0.81–1.16]	0.75	0.98	[0.80–1.20]	0.85
**Complicated or apparently random patterns**	1.1	[0.78–1.57]	0.58	1.05	[0.72–1.54]	0.80
	n = 341			n = 335		

RRR= Relative Risk Ratio; 95% CI= 95% Confidence Interval

Reference category is 'no change'

Maximally adjusted models were controlled for: gender, age, education level, the availability of (free) parking at work, housing tenure, presence of children in the household, moving working or residential location and residential settlement size.

An example of how the results could be interpreted: Individuals who live 4 km instead of 9 km away from the guided busway (or any other transformation that corresponds with a one-point increase in the exposure measure) are 12% more likely to have a travel behaviour pattern that can be classified as a ‘partial modal shift’ than a pattern that can be classified as ‘no change’. This effect is non-significant at a 95% confidence level.

## 4 Discussion

We characterised patterns of change in commuting behaviour over time, and tested whether exposure to a transport infrastructural intervention was associated with certain behaviour patterns, analysing a four-year cohort within a quasi-experimental study. Although previous analyses have connected exposure to the intervention with a higher likelihood of an increase in active travel and a decrease in car use (Panter et al., 2016, Heinen et al., 2015), in the present analysis, we did not find a significant association between the level of exposure to the intervention (residential proximity to the new infrastructure) and specific modal shifts, nor did we find evidence that exposure to the intervention was associated with belonging to a group that showed a full or partial modal shift. It therefore appears presumptuous to conclude that individuals who were more exposed to the intervention were more likely to change their travel behaviour by making a full shift from a car to a bicycle. An alternative explanation for the non-significant association may be the time between the intervention and last wave of data collection. Recently intervention studies have shown that new infrastructure may result in travel behaviour change. However, given the limited number of such studies, it has not been determined how long it takes before a full or partial shift may occur. In the study of Goodman et al. (2014), the intervention did not have a significant effect after one year, but after two years, exposure to the intervention predicted changes in travel behaviour. Therefore, future studies collecting follow-up data for a longer period after the intervention may result in additional insights.

The exploration of the patterns of mode choice revealed five groups in the cohort:

• *Group 1- No changes*: The individuals in this group showed similar behaviour over all four waves. Their stable behaviour comprised one mode or combination of modes only, or a stable day-to-day variation in the transport modes used on the commute.
• *Group 2- A full modal shift:* This group contained individuals who changed their commute mode choice over time.
• *Group 3- A partial modal shift*: The individuals in this group showed changes in their commute mode choice, but only for a proportion of their trips.
• *Group 4- Non-stable but patterned behaviour:* Individuals in this group showed commute behaviour that changed between waves and could not be classified as modal shifts. However, the behaviour pattern showed similarities between the waves.
• *Group 5- Complicated or apparently random patterns:* The final group contains individuals who had a highly variable pattern of commute mode choice, which appeared almost random.

The ‘no changes’ group may often be described as containing individuals who may have habitual behaviour, which have often been recognized as an important predictor of behaviour (e.g. Verplanken and Orbell, 2003; Verplanken et al., 1998). Habit in psychological literature is often understood as an automatic behaviour. However, in travel behaviour research, a less strict definition is sometimes applied, such as the consistency or frequency of use or number of trips made by a certain mode (Verplanken, 2006). Our findings partly corroborate the hypothesis that individuals may have habitual behaviour that is unlikely to change, in our case even when exposed to an intervention. However, a large proportion of our respondents did not show habitual behaviour, and even within the ‘no changes’ group, a subgroup was present comprised of individuals who used different modes for different commute trips. This may imply that many individuals do not have habitual behaviour following the psychological definition, i.e. their behaviour is not automatic.

A modal shift, preferably a full modal shift, has been a policy aim of many local and national governments. Our analyses revealed that a relatively small proportion of the cohort showed a full modal shift. This is noteworthy given the significant effect found of the intervention on changes in individual travel behaviour (Heinen et al., 2015, Panter et al., 2016). Our analyses showed that partial shifts were more common. This corresponds with recent findings on car use (Chatterjee et al., 2016) and corroborates the postulate that policies might more realistically target initial partial shifts, which are increasingly acknowledged as a potential first step of a fuller modal shift. A partial modal shift is often associated with increasing the variability of the modal mix, sometimes called multimodality (e.g. Jones and Clarke, 1988; Huff and Hanson, 1986; Kuhnimhof, 2009; Nobis, 2007). Recent research has revealed that multimodality is associated with higher levels of change (Kroesen, 2014, Heinen and Ogilvie, 2016). Additionally, in our cohort, we observed that some individuals first showed a partial shift, before ultimately shifting their mode choice entirely. Moreover, within the partial shift group, a larger tendency could be observed towards more variable travel behaviour.

It is important to acknowledge that more than a third of the individuals in our cohort belonged to either the ‘non-stable but patterned behaviour’ or the ‘complicated or apparently random patterns’ groups. This shows that a large level of variability in behaviour is present, without clearly distinguishable shifts.

### 4.1 Implications

Our analyses revealed that many individuals showed variation in their mode choices. We can see in the patterns in the use of car, active travel, and public transport in particular that although a large proportion of car and bicycle users use only that mode (for the entire trip and every day), a significant share of our cohort shows day-to-day variability. This observation has implications for research on mode choice. Questions such as ‘How do you usually travel to work?’ are unable to fully capture travel behaviour and are likely to overestimate the use of the main mode of the modal mix. Similarly, questions that ask about certain journeys, such as ‘How did you travel to work yesterday?’ may have a bias towards the main modes of transport. Although it is often assumed that the random variability within individuals will be balanced on a population level, our findings do not support this as our data revealed that active and public transport commuters appear more likely to use multiple ways to travel for commuting purposes, and these modes are therefore more likely to be underestimated.

Secondly, our description of the patterning of individual travel behaviour has implications for how best to measure behaviour change. The patterning, as presented, did not only show variance in mode choice within a week for many participants, it also showed changes over time. We therefore need to be prudent in labelling measured differences between time points as change, and should develop data collection strategies that allow for differentiation between change and (random) variability, for which measuring travel behaviour for a period of time (as opposed to a single trip or day) appears to be essential. Intervention studies may provide us with the necessary insights for disentangling behaviour change and behavioural variability.

Thirdly, more than one third of the individuals in our cohort belonged to either the ‘non-stable but patterned behaviour’ or the ‘complicated or apparently random patterns’ groups. It is not entirely clear what factors underlie such patterns. Individuals with these patterns may have less fixed or more complicated lives and correspondingly more flexible uses of various commute modes to enable participation in a variety of activities (e.g. social, cultural or child-care-related) before and after work. Another explanation may be that work characteristics, such varying working locations either day-to-day or year-to-year, may increase the level of variability. Alternatively, a given surveyed week may be—from the individual perspective—an exceptional one in terms of travel behaviour. Nevertheless, our explorations show that high levels of variability are unexceptional, and perhaps even more the norm than stable travel behaviour. For future interventions, it is important to understand the causes and consequences of these high levels of variability. The size of these groups may imply that for a large share of the population, a full modal shift may be unlikely. To effectuate a shift towards healthier and more sustainable form of transport, it is therefore important to know which part of their travel behaviour may be changeable. A contrasting conclusion may also be drawn: that high levels of variability indicate that a large share of the population is potentially open to behaviour change, as research has found that higher levels of multimodality are associated with an increased likelihood to change travel behaviour when exposed to an intervention (Heinen and Ogilvie, 2016). For example, the use of a greater number of transport modes may result in higher self-efficacy to use a wide variety of modes. This may make such individuals more open to an intervention aiming for a modal shift if such an intervention improves the ease with which a certain mode can be used.

Finally, we recommend longer-term evaluations of interventions. Our exploration of the patterns of change revealed that full modal shifts were sometimes preceded by partial modal shifts. Although our data did not allow us to investigate whether respondents who initially showed a partial modal shift will continue to change their behaviour and will eventually show a full modal shift, or whether they will return to their initial or even to a third pattern of behaviour, explorations of patterns of travel behaviour over a longer time frame may reveal which patterns may be most common. This will contribute to a fuller understanding of the intervention effects on travel behaviour change.

Although policies may ultimately aim to create a healthful and sustainable society for which full modal shifts towards active travel may be required, our results indicate that partial modal splits were more prevalent. This could indicate that policies perhaps should initially aim for a shift in the desired direction and not aim for a full modal shift immediately.

### 4.2 Strengths and limitations

This study has provided an extensive exploration of individual patterns of travel behaviour and travel behaviour change. Key strengths include the quasi-experimental study design, which allowed us to investigate behaviour change in the context of an intervention. Secondly, we explored patterns of travel behaviour over a four-year period, each period consisting on one-week commute data at a stage level. This allowed for a detailed exploration of changes and variability in mode choice over time. Our analyses aimed to reveal patterns (of change) in individual mode choice over time. Some commonly used statistical techniques such as cluster analysis or latent class analysis might also have been applied in order to group individuals by either observed or unobserved characteristics. However these techniques are not very suitable for grouping individuals by weekly mode choice patterns over four years. We therefore used a combination of visual explorations and simpler statistical analyses. However, this study has several shortcomings. First, travel behaviour was self-reported, which may have resulted in intentional or unintentional misreporting. Second, our sample was not representative for Cambridge. Third and most importantly, we had only a relatively small sample size. Although we have found significant associations between exposure and changes in travel behaviour in other analyses, perhaps due to the sample size, we were unable to find any significant associations between exposure to the intervention and specific modal shifts in this analysis.

## 5 Conclusion

This study has provided an in-depth exploration of patterns of travel behaviour and travel behaviour change. Five groups of patterns of change were found via in-depth explorations: (1) no changes, (2) a full modal shift, (3) a partial modal shift, (4) non-stable but patterned behaviour, and (5) complicated or apparently random patterns. We did not find evidence that exposure to the intervention was associated with specific modal shifts, or with belonging to any of the groups of patterns of change. These findings indicate that the intervention did not result in a specific pattern of behavioural change. This deeper understanding of mode choice changes may lead to the ensuing increased capability to predict consequent effects, e.g. air pollution, congestion, and physical activity, as well as providing insight into the potential modal shift impacts of comparable interventions in the future, thereby contributing to improving population health and well-being.

## Appendix A Overview of car use

Each horizontal line is an individual. Each trip is one square and the length of the line corresponds with the number of commute trips made over one week. The waves are separated by vertical black lines.

Dark red = the sole use of a car in a trip; light red = the partial use of a car.

The white colour indicates no trip was made. The grey colour indicates that the trip did not involve any car use. The visualisations are clustered by intensity of use. Individuals who always only travelled by car are positioned at the top, followed by individuals who always used the car for part of their journey. The individuals who sometimes travelled by car are clustered by type of behaviour, i.e. the use of the car for the entire trip or part of the trip. Individuals who never use the car are positioned at the bottom.

## Appendix B Overview of use of active travel modes

Each horizontal line is an individual. Each trip is one square. The waves are separated by vertical black lines.

Dark red = the sole use of a bicycle in a trip and the length of the line corresponds with the number of commute trips made over one week; light red = the partial use of a bicycle in a trip; dark blue = the sole use of walking; light blue = partial walking in a trip; pink = the use of both walking and cycling in a trip. The white colour indicates no trip was made. The grey colour indicates that the trip did not involve any walking or cycling. The visualisations are clustered by intensity of use.

## Appendix C Overview of public transport use

Each horizontal line is an individual. Each trip is one square. The waves are separated by vertical black lines.

Dark red = the sole use of a train in a trip and the length of the line corresponds with the number of commute trips made over one week; dark orange = the sole use of a bus in a trip; light red = the partial use of a train; light orange = the partial use of a bus. The white colour indicates no trip was made. The grey colour indicates that the trip did not involve any public transport. The visualisations are clustered by intensity of use.
